# Occurrence and diversity pattern of CRISPR-Cas systems in *Acetobacter* genus provides insights on adaptive defense mechanisms against to invasive DNAs

**DOI:** 10.3389/fmicb.2024.1357156

**Published:** 2024-07-11

**Authors:** Sara Ghaffarian, Bahman Panahi

**Affiliations:** ^1^Department of Cellular and Molecular Biology, Faculty of Sciences, Azarbaijan Shahid Madani University, Tabriz, Iran; ^2^Department of Genomics, Branch for Northwest & West region, Agricultural Biotechnology Research Institute of Iran (ABRII), Agricultural Research, Education and Extension Organization (AREEO), Tabriz, Iran

**Keywords:** Acetobacter, CRISPR-Cas system, diversity, evolution, adaptive defense mechanisms

## Abstract

The *Acetobacter* genus is primarily known for its significance in acetic acid production and its application in various industrial processes. This study aimed to shed light on the prevalence, diversity, and functional implications of CRISPR-Cas systems in the *Acetobacter* genus using a genome mining approach. The investigation analyzed the CRISPR-Cas architectures and components of 34 Acetobacter species, as well as the evolutionary strategies employed by these bacteria in response to phage invasion and foreign DNA. Furthermore, phylogenetic analysis based on CAS1 protein sequences was performed to gain insights into the evolutionary relationships among Acetobacter strains, with an emphasis on the potential of this protein for genotyping purposes. The results showed that 15 species had orphan, while20 species had complete CRISPR-Cas systems, resulting in an occurrence rate of 38% for complete systems in Acetobacter strains. The predicted complete CRISPR-Cas systems were categorized into I-C, I-F, I-E, and II-C subtypes, with subtype I-E being the most prevalent in Acetobacter. Additionally, spacer homology analysis revealed against such the dynamic interaction between Acetobacter strains and foreign invasive DNAs, emphasizing the pivotal role of CRISPR-Cas systems in defending against such invasions. Furthermore, the investigation of the secondary structures of CRISPR arrays revealed the conserved patterns within subtypes despite variations in repeat sequences. The exploration of protospacer adjacent motifs (PAMs) identified distinct recognition motifs in the flanking regions of protospacers. In conclusion, this research not only contributes to the growing body of knowledge on CRISPR-Cas systems but also establishes a foundation for future studies on the adaptive defense mechanisms of Acetobacter. The findings provide valuable insights into the intricate interplay between bacteria and phages, with implications for industrial applications and potential biotechnological advancements.

## Introduction

Acetobacter, the largest group of bacteria in the Acetobacteraceae family, is a genus of acetic acid bacteria known for its industrial significance and diverse ecological distribution (Qiu et al., 2021). This genus consists of 33 validly published species that display gram-negative, obligate aerobic, and catalase-positive traits. Acetobacter species have a wide ecological niches, commonly found in hot and humid areas, fruits, flowers, soil, intestines, and vinegar (Matsushita et al., 2016). Recent taxonomic advancements have resulted in the classification of these bacteria into 19 genera, highlighting their genetic and phenotypic diversity (Lynch et al., 2019).

The metabolic pathways of Acetobacter, including ethanol oxidation respiratory chain, tricarboxylic acid cycle, pyruvate metabolism, and pentose phosphate, enable them to produce large quantities of acetic acid, a key industrial product (Raspor and Goranovič, 2008). With an optimal temperature range of 25–30°C for acetic acid production and high acid resistance, Acetobacter species are essential microorganisms in industrial settings, renowned for their efficient utilization of biomass. They find wide application in vinegar and fruit vinegar production, gluconic acid products, and biofuel cell development, underscoring their economic and industrial significance (Lynch et al., 2019).

Additionally, specific strains of Acetobacter have been identifies as to facilitating plant growth and contributing to the global nitrogen cycle, further highlighting their ecological impact (Krotzky and Werner, 1987). However, the presence of bacteriophages, or phages, poses a significant threat to Acetobacter activity in various industrial processes, including fermented dairy product production (Garneau and Moineau, 2011). Phage’s reduce acid production and alter the taste and texture of the final product, highlighting the importance of studying and understanding the relationship between phages and Acetobacter hosts (Nami et al., 2021, 2023; Panahi et al., 2022; Rostampour et al., 2022; Ghaffarian and Panahi, 2023.

Clustered regularly interspaced short palindromic repeats (CRISPR) and related Cas proteins serve as a defense system against phages or any foreign DNA in most bacteria and archaea (Nami et al., 2021). This adaptive immune system is characterized by short nucleotide repeats interspersed with spacers. When a virus attacks the bacteria, a portion of the virus genome is inserted into the conserved repeats of the CRISPR array. Transcription of the CRISPR repetitive sequences and non-repetitive spacer sequences leads to the creation of a contiguous single-stranded RNA sequence (pre-crRNA), which, then subjected to the processing, leading the production of mature crRNA (Panahi et al., 2022).

Recent advancements in next-generation sequencing technologies and genome mining approaches have facilitated comprehensive genome-wide analysis of the occurrence and diversity of CRISPR-Cas systems (Nami et al., 2023). As a results, a comprehensive database of potential CRISPR arrays has been developed, providing significant insight into the adaptive defense mechanisms employed by bacteria and archaea against phages and other invasive DNA forms (Makarova and Koonin, 2015).

The study of CRISPR-Cas systems in bacteria and archaea has revealed remarkable complexity and diversity, leading to the categorization of CRISPR-Cas systems into distinct types and subtypes (Liu et al., 2020). These systems, represented by CRISPR-Cas modules, serve as adaptive antivirus immunity mechanisms and are found in most archaea and many bacteria (Medina-Aparicio et al., 2018).

Furthermore, researchers are investigating the potential of these identified systems in genotyping and the development of CRISPR applications (Panahi et al., 2022). The increasing availability of genomes also present an opportunity to re-evaluate the CRISPR-Cas systems of recently submitted strains. Consequently, a comprehensive genome-mining approach was employed in this study to examine the diversity, occurrence, and evolution of the CRISPR-Cas system in Acetobacter strains. The findings have implications for industrial applications and potential advancement in biotechnology.

## Materials and methods

### Data collection and CRISPR/Cas system identification

The genome sequences of 34 Acetobacter species were obtained from the NCBI database.^1^ For the prediction of CRISPR-Cas systems, the CRISPRone web server^2^ was utilized. Only CRISPR loci that contained the complete sets of associated proteins for each subtype were included in the subsequent analysis. Furthermore, the direction of CRISPR/Cas arrays, the number and sequence of spacers, the length and sequence of repeats, and the designation of subtypes were determined using BLST software, as described in Panahi et al. (2022).

### Secondary structure prediction of repeats

The secondary structures of RNA were predicted using the minimum free energy (MFE) algorithm provided by the RNA fold Web server (Gruber et al., 2008). The default parameters were used for the prediction. The mentioned server incorporates a partition function implementation that calculates the probabilities of based pairings and allows for RNA folding (Hofacker, 2003).

### CRISPR-Cas system characterization

CRISPR arrays repeats and spacers were extracted and visually represented using CRISPRviz tools. CRISPRviz compares the nucleotide sequences of spacers and repeats and assigns a representative color to each element that carries a meaningful interpretation (Nethery and Barrangou, 2019). Furthermore, the evolutionary relationships of the identified systems were analyzed under the influence of addition and deletion pressure.

### Analysis of protospacer adjacent motif

Protospacers were identified using the BLAST program, as described in (Nami et al., 2023). Protospacers with more than 85% identity and less than 3 mismatches were selected for further analysis. To determine the protospacer adjacent motif (PAM), the 10 nucleotide flanking regions on the 5′ and 3′ ends of the protospacer sequences were aligned and representative logo were generated suing the WebLogo server.^3^

### Phylogenetic analyses

To examine the evolutionary relationships among the identified CRISPR-Cas systems in Acetobacter species, the amino acid sequences of the predicted systems CAS1 protein were utilized for constructing a phylogenetic tree. Initially, the sequences were aligned using CLUSTAL X software. Subsequently, the Neighbor Joining algorithm was employed to calculate the genetic distance between the Cas1 proteins in the identified systems. The resulting matrices were then utilized in the construction of phylogeny tree using MEGA software (version 11).

## Results and discussion

### Occurrence of CRISPR-Cas systems in *Acetobacter* genus

In this study available genomes of 33 Acetobacter species were downloaded from the NCBI GenBank database and analyzed for the occurrence and diversity of CRISPR/Cas systems. The studied strains were categorized into two groups based on their CRISPR/Cas systems. CRISPR/Cas systems that contained repeat/spacer arrays flanked by a series of Cas genes were classified as complete and strains that did not contain Cas genes recognized as orphan CRISPR/Cas systems. According to this classification, strains belonging to the *A. fabarum*, *A. conturbans*, *A. oeni*, *A. musti*, *A. suratthaniensis*, *A. sacchari*, *A. fallax*, *A. garciniae*, *A*. *ghanensis*, *A. orleanensis*, *A. okinawensis*, *A*. *cerevisiae*, *A*. *papayae*, *A. peroxydans,* and *A. lovaniensis* species did not have any complete CRISPR/Cas systems in their genomes. On the other hand, 138 strains belonging to 20 species had complete CRISPR/Cas systems in their genomes (Table 1). In total, the occurrence rate of complete CRISPR/Cas systems in Acetobacter strains was about 38% which was lower than the estimated prevalence in other bacteria (45%) (Grissa et al., 2007). The occurance rate for *Clostridium perfringens*, *Lactobacillus casei*, *Lactobacillus brevis*, *Lactobacillus johnsonii,* Leuconostoc, and Bifidobacterium were 53.15, 39, 27, 22, 35, and 77%, respectively, (Briner et al., 2015; Long et al., 2019; Yang et al., 2020; Panahi et al., 2022; Ghaffarian and Panahi, 2023; Nami et al., 2023). Remarkably, *A. pasteurianus* and *A. malorum* species had the highest and lowest rate of complete CRISPR/Cas occurrence, respectively. Overall, the results indicated that the estimated occurrence rates of complete CRISPR/Cas systems in Acetobacter was 57.97%, which was significantly higher than the previously reported rate of CRISPR/Cas systems occurrence in other bacteria. The diverse rates of CRISPR/Cas systems occurrence suggests that bacteria have adapted to a wide range of environmental challenges, including exposure to various invasive genetic elements.

**Table 1:** Clustered regularly interspaced short palindromic repeats/CRISPR-associated system in *L. brevis* strains with confirmed CRISPR array.

Species	Strains	CRISPR/Cas type	Direction	Species	Strains	CRISPR/Cas type	Direction
*A. ascendens*	LMG 1591	I-F	Positive	*A. indonesiensis*	5H-1	I-E	Positive
*A. estunensis*	LMG 1626	I-E	Positive		DmL_051	I-E	Positive
*A. farinalis*	LMG 26772	I-E	Positive		NBRC 16471	I-E	Negative
*A. lambici*	LMG 27439	I-E	Positive		R-82819	I-E	Negative
*A. malorum*	LMG 1746	I-F	Negative		UGAI07	I-E	Positive
*A. thailandicus*	Dm-29	I-F	Positive	*A. tropicalis*	DmW_042	I-C	Positive
*A. cibinongensis*	4H-1	I-F	Negative		CS_006_W138	I-C	Negative
	NBRC	I-F	Positive		CS_006_W139	I-C	Positive
*A. nitrogenifigens*	DSM 23921	Universal	Negative		DmCS_006	I-C	Positive
	NBRC 105050	Universal	Negative		DmPark25_167	I-C	Positive
*A. oryzifermentans*	SLV-7	I-E	Positive		LMG 1663	I-C	Negative
	dm	I-E	Negative		LMG 19825	I-C	Negative
*A. oryzoeni*	B6	I-E	Negative		NBRC 16470	I-C	Positive
	R-80287	I-E	Positive		NBRC 16470 (2)	I-C	Negative
*A. sicerae*	DmPark20a_162	I-E	Negative	*A. pasteurianus*	IFO 3283–01-42C	I-E	Positive
	LMG 1531	I-E	Negative		IFO 3283–03	I-E	Positive
*A. persici*	Dm-46	I-C	Negative		CICC 22518	I-F	Positive
	JCM 25330	I-C	Negative		IFO 3283–07	I-E	Positive
	TMW2.1084	I-F	Positive		IFO 3283–01	I-E	Positive
*A. senegalensis*	A3	I-E	Negative		IFO 3283–12	I-E	Negative
	GYC10	I-E	Positive		IFO 3283–22	I-E	Positive
	GYC12	I-E	Positive		IFO 3283–26	I-E	Positive
	GYC19	I-E	Positive		IFO 3283–32	I-E	Positive
	MRS7	I-E	Negative		NBRC 3284	I-E	Positive
	R-80417	Universal	Positive		NBRC 3277	I-F	Negative
*A. aceti*	ATCC 23746	II-C	Positive		NBRC 3278	I-F	Positive
	DSM 3508	II-C	Positive		NBRC 3279	I-E	Negative
	JCM20276	II-C	Positive		NBRC 3280	I-F	Positive
	NBRC 3281 C1	I-E	Negative		SRCM100623	I-E	Positive
	NBRC 3281 C2	I-E	Positive		SRCM101468	I-E	Positive
	NBRC 14818	II-C	Negative		UBA5418	I-E	Negative
	NBRC 14818 (2)	II-C	Negative	*A. pomorum*	BDGP5	I-C	Negative
	NRIC 0242	II-C	Negative		CS_004_W120	I-C	Negative
*A. orientalis*	DmW_045	I-F	Negative		CS_004_W121	I-C	Positive
	DsW_061	I-F	Negative		CS_004_W122	I-C	Positive
	R-83288	I-F	Positive		DM001	I-C	Negative
	DmW_045	I-F	Negative		DmCS_004	I-C	Positive
*A. syzygii*	KR-1	I-C	Negative		LHT 2458	I-F	Positive
	KR-2	I-C	Negative		SH	I-E	Positive

### Diversity pattern of CRISPR-Cas systems

**Figure 1:**
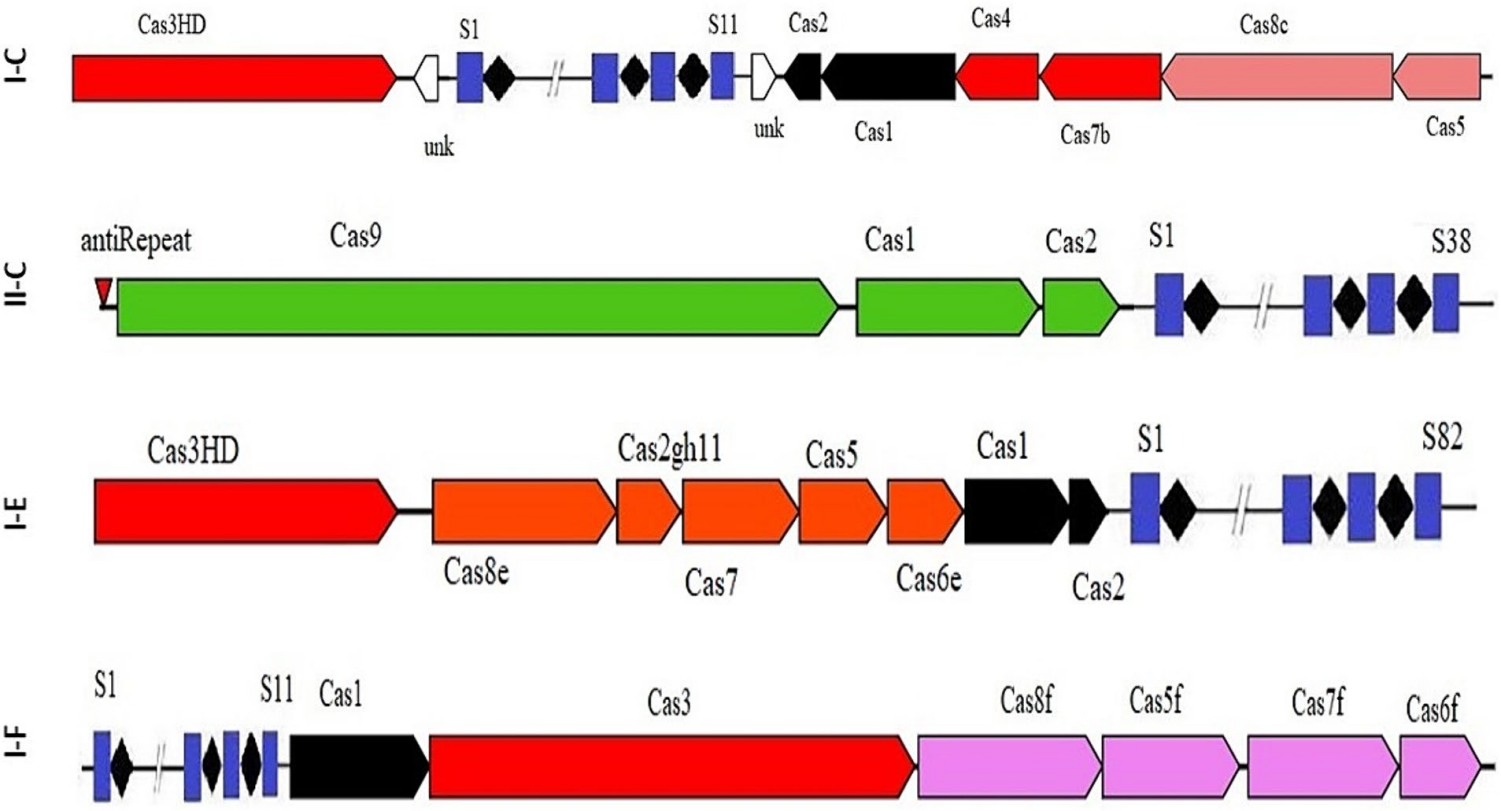
Schematic diagram of complete CRISPR-Cas array in subtypes II-C; I-C; I-E; and I-F.

The results indicated that 67 strains had the CRISPR-Cas systems of subtype I, specifically belonging to subtypes I-C, I-F, or I-E. Additionally, all identified CRISPR-Cas subtype II systems were classified as II-C (Figure 1). Our survey also revealed that the strains included in the study did not have subtype III CRISPR-Cas systems in their genomes. Consistent with our findings, Briner et al. (2015) also reported the absence of subtype III CRISPR-Cas systems in *Bifidobacterium* genus.

The occurrence rate of CRISPR-Cas systems in different species and strains of Acetobacter showed that the CRISPR-Cas subtype I-E (52.24%) and I-F (19.4%) have the highest and lowest prevalence, among different subtype I, respectively. Additionally, the occurrence rate of subtype II-C, the only predicted CRISPR-Cas subtype II in Acetobacter, is only 8% of total.

Some bacterial genomes have a high occurrence rate of CRISPR-Cas systems with 77% of the analyzed genomes containing these CRISPR-Cas systems (Briner et al., 2015). The distribution and prevalence of CRISPR-Cas systems are influenced not only by genetic factors, but also by the environmental conditions in which the strains originated. It has been demonstrated that strains isolated from high temperature conditions have a higher occurrence rate of CRISPR- occurrence/Cas in their genome (Lan et al., 2022).

Among different species of Acetobacter, *A. pomorum* had three different CRISPR-Cas subtypes, namely I-C, I-F, and I-E, in its genome, making it the most diverse species in terms of CRISPR system subtypes (Table 1). Consistent with our findings, Briner et al. (2015) reported that Bifidobacterium has CRISPR-Cas systems belonging to the subtypes I-C, I-E, I-U, II-C, and II-A in its genome.

**Figure 2:**
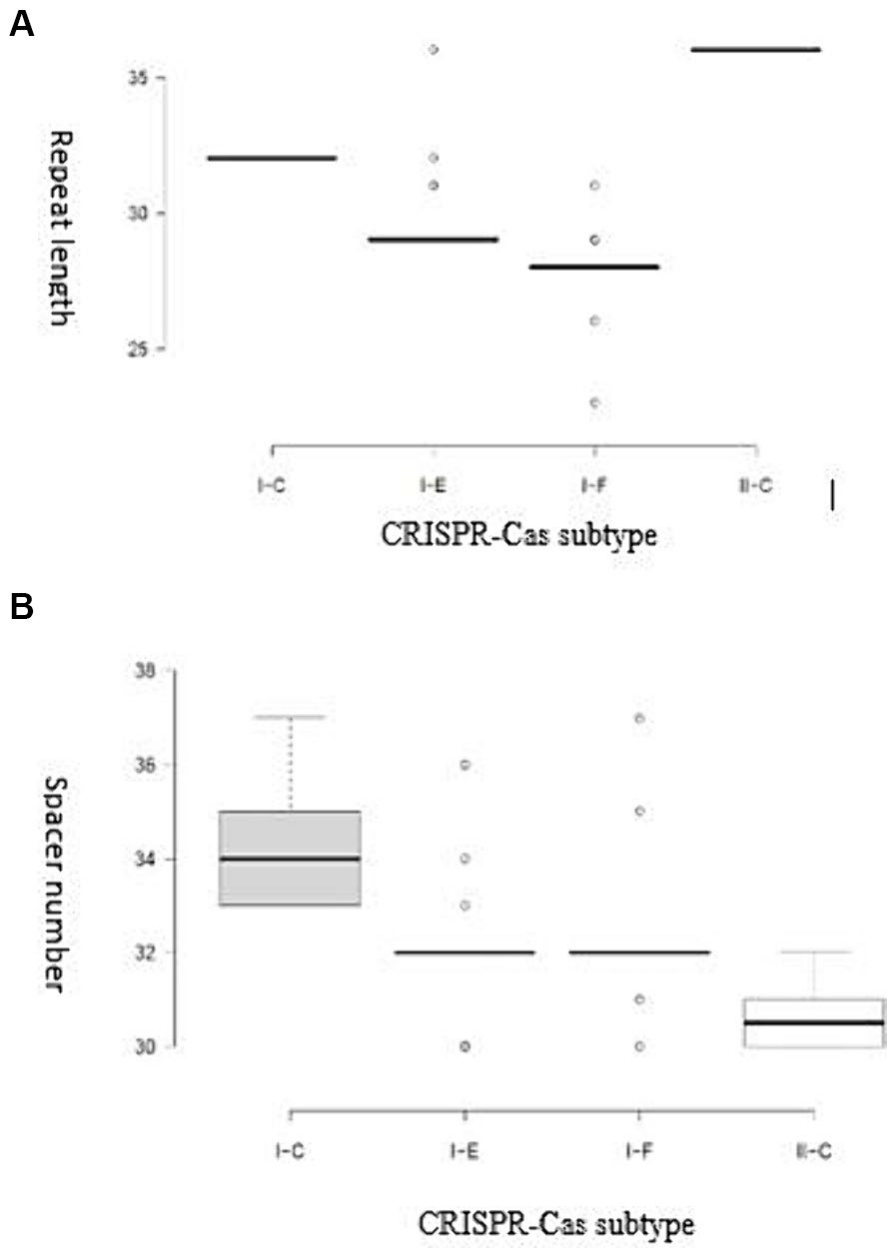
**(A)** Distribution of the repeat length for each CRISPR-Cas subtype; **(B)** Distribution of spacer numbers for each CRISPR-Cas subtype. Trans-activating CRISPR (tracr) RNA for subtype II systems is shown in yellow.

It has been demonstrated that the Cas genes can be categorized into four modules based on their functional impacts: adaptation, expression, interference, and signal transduction/ancillary during immunity modulation against invasive DNAs. The results of our study showed that each subtype harbor unique characteristics within each module in terms of Cas proteins diversity, highlighting the structural variation of CRISPR-Cas system associated proteins and their relevant implications (Makarova and Koonin, 2015). It has been shown that the diversity of components in the functional modules of the CRISPR/Cas complex enables the system to identify and adjust to a broad spectrum of invading components (Rostampour et al., 2022; Ghaffarian and Panahi, 2023). A detailed understanding of the structural features is crucial for comprehending the function of CRISPR/Cas systems in the studied strains. Variations in the structure of CRISPR arrays result in the acquisition of new and different spacers and repeat elements. The analysis results showed that the average length of repeats for the I-C, I-F, I-E, and II-C subtypes were 32, 27.94, 29.24, and 36 bp, respectively. The spacer sequences, responsible for recognizing foreign DNA, in the II-C, I-C, I-E, and I-F subtypes were 30–32 bp, 33–37 bp, 30–36 bp, and 30–37 bp in length, respectively (Figure 2).

**Figure 3:**
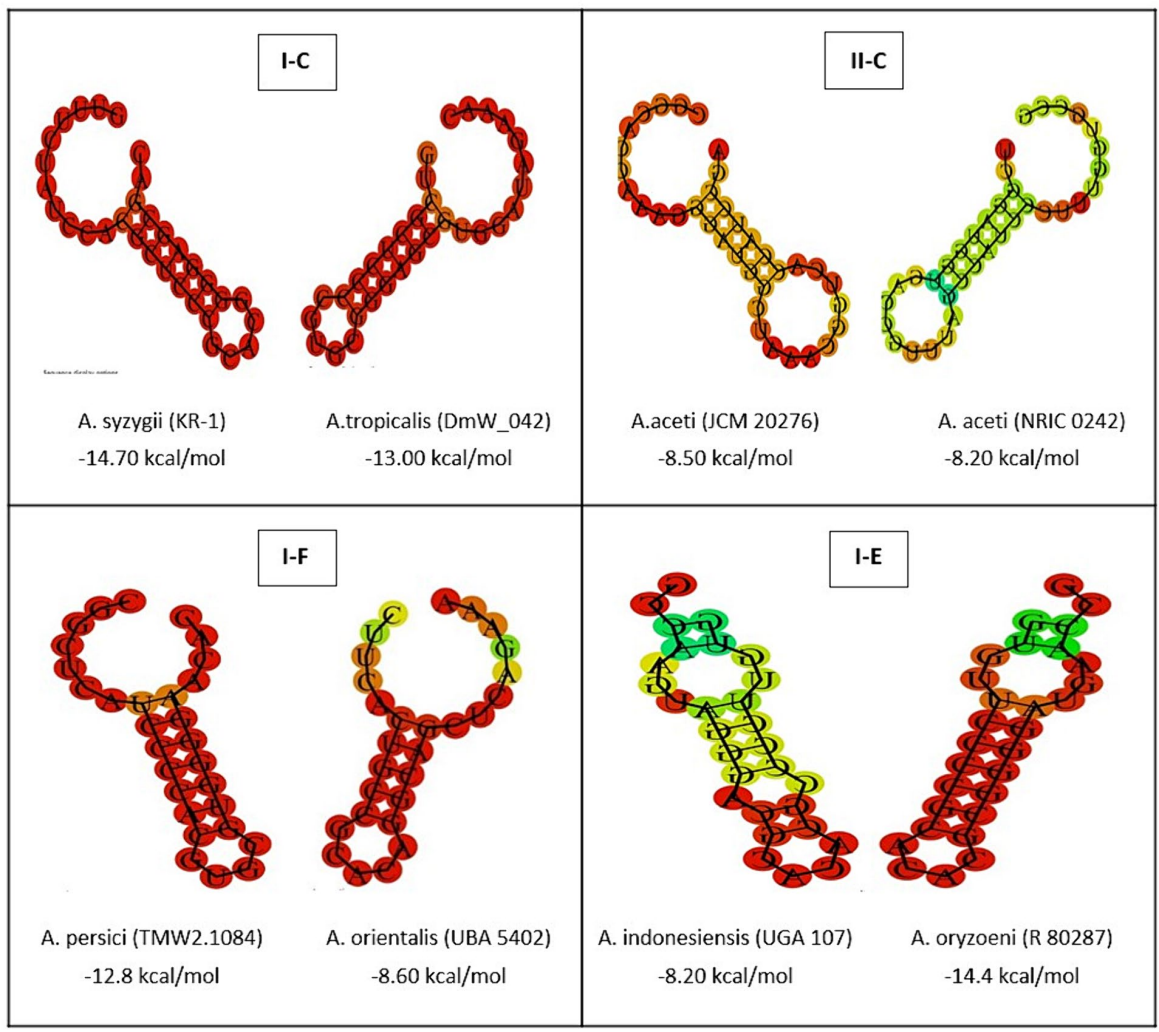
The prediction of consensus direct repeat secondary structure and corresponding MFE values in subtypes II-C; I-C; I-E; and I-F. According to the default parameters adjusted by the system, red and green regions indicated the high and low probability of conformation in each structure.

The complex relationships between the structural characteristics of direct consensus repeats within CRISPR arrays is crucial for understanding the function of CRISPR-Cas systems in the investigated strains. Through a structural analysis, this study revealed valuable insight into of repeat sequences across different CRISPR subtypes (I-C, II-C, I-F, and I-E) (Figure 2). By identifying of palindromes, inverted repeats, and stable secondary structures, such as stem-loops, provides new insights into the underlying molecular architecture of array elements in the Acetobacter genera. Moreover, the current study highlights how specific features, such as stem length, GC pair content, and the presence of A-U pairs, contribute to the stability and function of CRISPR-Cas systems. According previous findings, stem length and GC pair content determine the stability of the structure, with longer stems and higher CG pairs contributing to the higher stability (Yang et al., 2020). In comparison with the subtype I (C, F, and E) CRISPR systems, subtype II systems showed the lowest MFE and stability in the predicted secondary conformation of repeat sequence (Figure 3). The stability of the repeat conformation is closely linked to the efficiency of the CRISPR-Cas system in mediating interference against foreign nucleic acids. Unstable repeats may impede the formation of functional CRISPR RNA (crRNA) molecules or the proper assembly of Cas protein complexes, therefore, reducing the system’s interference capability and modulating immunity (Athukoralage et al., 2020). Moreover, unstable repeats are more susceptible to mutations, which can potentially impact the heritability and long-term functionality of the CRISPR-Cas system within microbial populations in different environments (Ghaffarian and Panahi, 2023).

### Protospacer adjacent motif prediction

The protospacer adjacent motif (PAM) is a critical component of CRISPR-Cas systems. It is a DNA sequence consisting of 2–6 base pairs located immediately after the DNA sequence targeted by the nuclease in the CRISPR bacterial adaptive immune system (Gleditzsch et al., 2019). The PAM is present in a component of the invading virus or plasmid, but not in the bacterial host genome, and therefore does not appear in the bacterial CRISPR locus. Cas9 cannot bind to or cleave the target DNA sequence if it is not followed by the PAM sequence (Liu et al., 2020). Moreover, the PAM is an essential targeting component that is distinguishes between bacterial self and non-self DNA, thereby protecting the CRISPR locus from being targeted and destroyed by the CRISPR-associated nuclease (Mojica et al., 2009). Therefore, the presence and diversity of PAM motifs have a significant implication for the function of CRISPR-Cas systems. Leenay and Beisel (2017) and Long et al. (2019) reported that the PAM for subtype I CRISPR-Cas system is located immediately at the 5′end of protospacers, whereas the PAM for the type II system is located at the 3′ end of protospacers.

**Figure 4:**
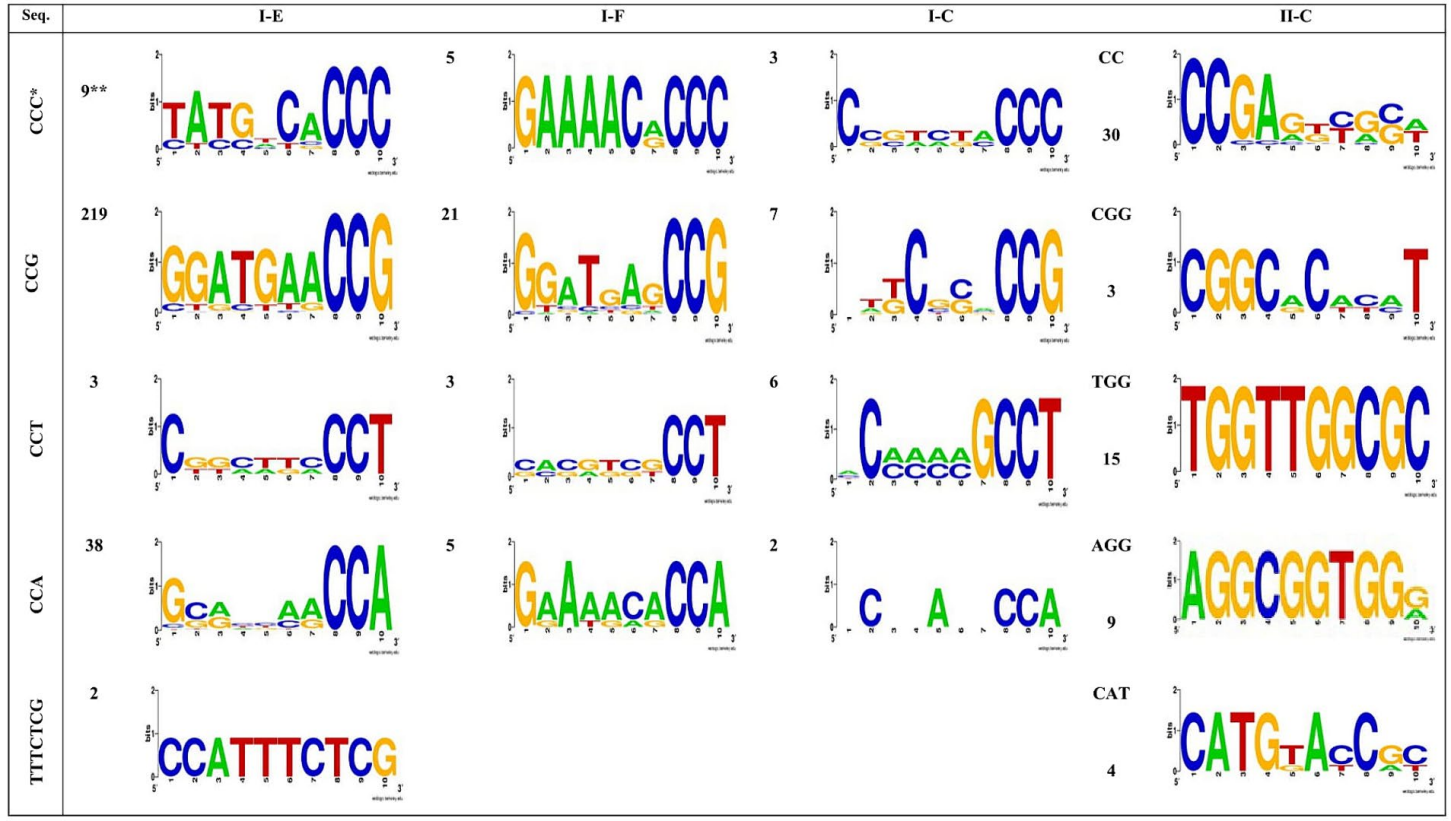
Predicted PAMs motifs in Acetobacter strain for strains which harbored the 5′ (I-C, I-E and I-F subtypes) and 3′ (II-C subtypes) flanking. * The height of each letter represents the conservation of that nucleotide at each position. ** The numbers next to the shapes, shows each motifs repeat number.

Figure 4 illustrates the identified PAM motifs for each predicted CRISPR-Cas system. It is evident that there are six types of PAM motifs, including 5’-CCG-3′, 5’-CCA-3′, 5’-CCC-3′, 5’-CCT-3′, 5’-TTTCTCG −3′, and 5′-TTTGTGG-3′ which are found flanking the 5′ end of protospacers in subtype I-E arrays. The I-E subtype exhibits the highest diversity in terms of PAM motif types and the highest number of identified PAMs flanking the 5′ end of protospacers. This subtype is confirmed to be the most active CRISPR-Cas system in the Acetobacter genus. Moreover, four PAM motifs, including 5’-CCG-3′, 5’-CCA-3′, 5’-CCC-3′, and 5’-CCT-3′, were identified in the 5′ end flanking regions of protospacers in subtype I-F and I-C arrays (Figure 4). Among the I- (E, C, and F) subtypes, 5’-CCG-3′ was the most frequent and serve as the most important functional PAM in the Acetobacter genus. The number and types of identified PAM motifs flanking the 3′ end of protospacers in all subtypes were higher than those present at the 5′ end (Figure 4). Subtype II-C exhibits the present of 3’-CGG-5′ and 3’-CC-5′ motifs.

#### Phylogeny analysis of *Acetobacter* genus strains

**Figure 5:**
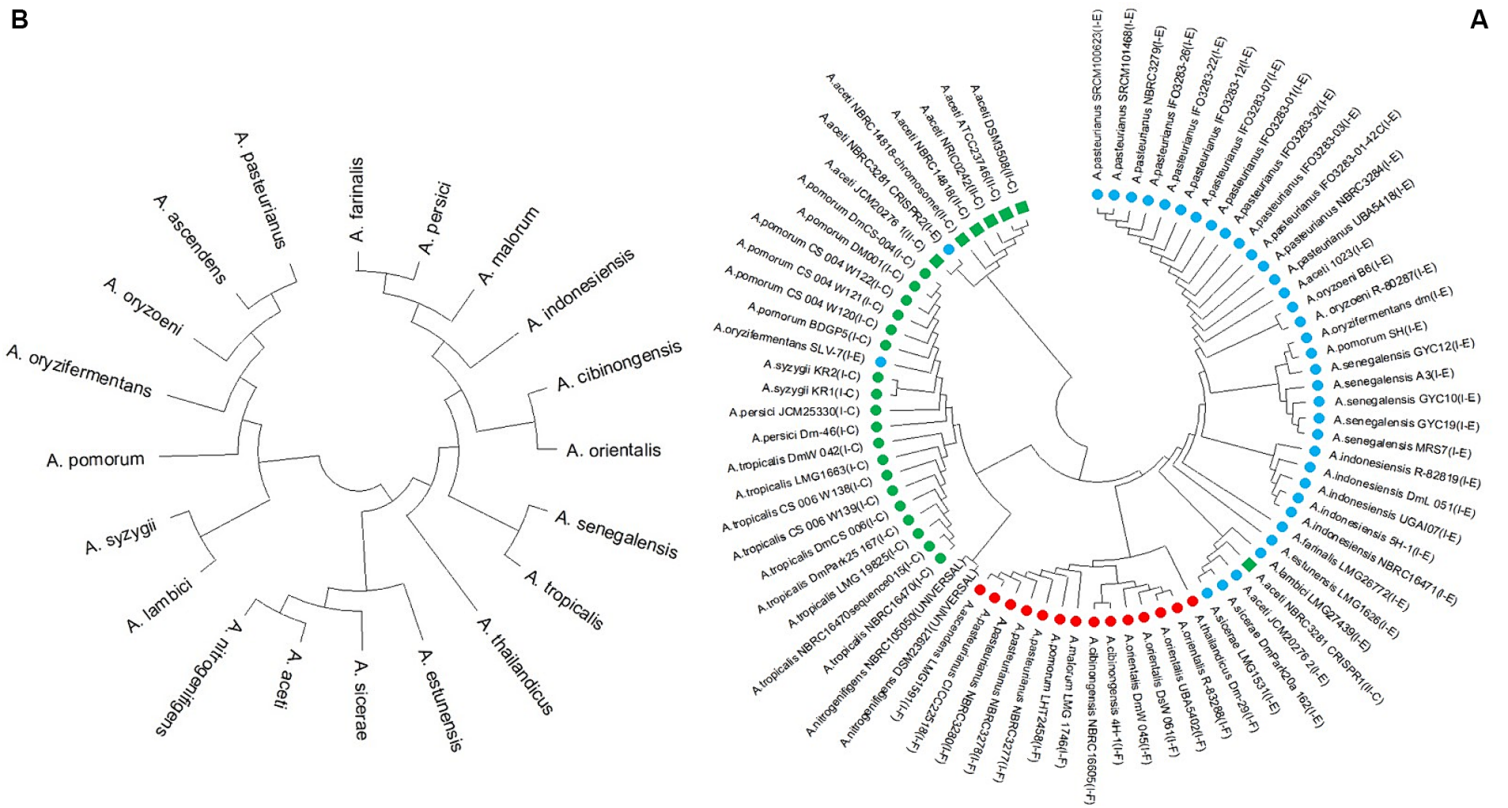
**(A)** Phylogeny tree based on Cas1 amino acid sequence and (Green box, green circle, blue circle, and red circle indicate the II-C, I-C, I-E, and I-F subtypes in Acetobacter strains, respectively) and **(B)** 16S rRNA nucleotide sequence.

Based on the multiple alignment of the amino acid sequence of Cas1 proteins, it is apparent that all II-C subtypes, except for NBRC 3281 of *A. aceti*, belong to a distinct group separate from the strains, which are all classified as subtype-I. The phylogeny analysis of the remain subtype-I strains reveals that they are clustered together in one group, with two subgroups. Strains of I-C subtype are grouped in one of these subgroups, while I-E and I-F strains form in the second subgroup, with independent branching. Furthermore, strains from the same species tend to cluster together in the same subgroup. Notably, the NBRC 3281 strain of *A. aceti*, which possesses two CRISPR systems (I-E and II-C), exhibits a distinct clustering pattern (Figure 5).

Additionally, the phylogenetic tree was constructed based on the 16S rRNA nucleotide sequence (Figure 5B). This analysis revealed a completely different grouping pattern compared to the Cas1-based phylogenetic tree. In the Cas1-based phylogeny, strains with similar CRISPR/Cas systems were grouped into the same subgroups, indicating that these protein sequences are highly conserved within subtypes of the CRISPR/Cas system. However, the grouping based on the 16S rRNA sequence is independent of the species phylogeny derived from the CRISPR/Cas system sequence (Figures 5A,B).

#### Spacerosome diversity, evolution, and targets

**Figure 6:**
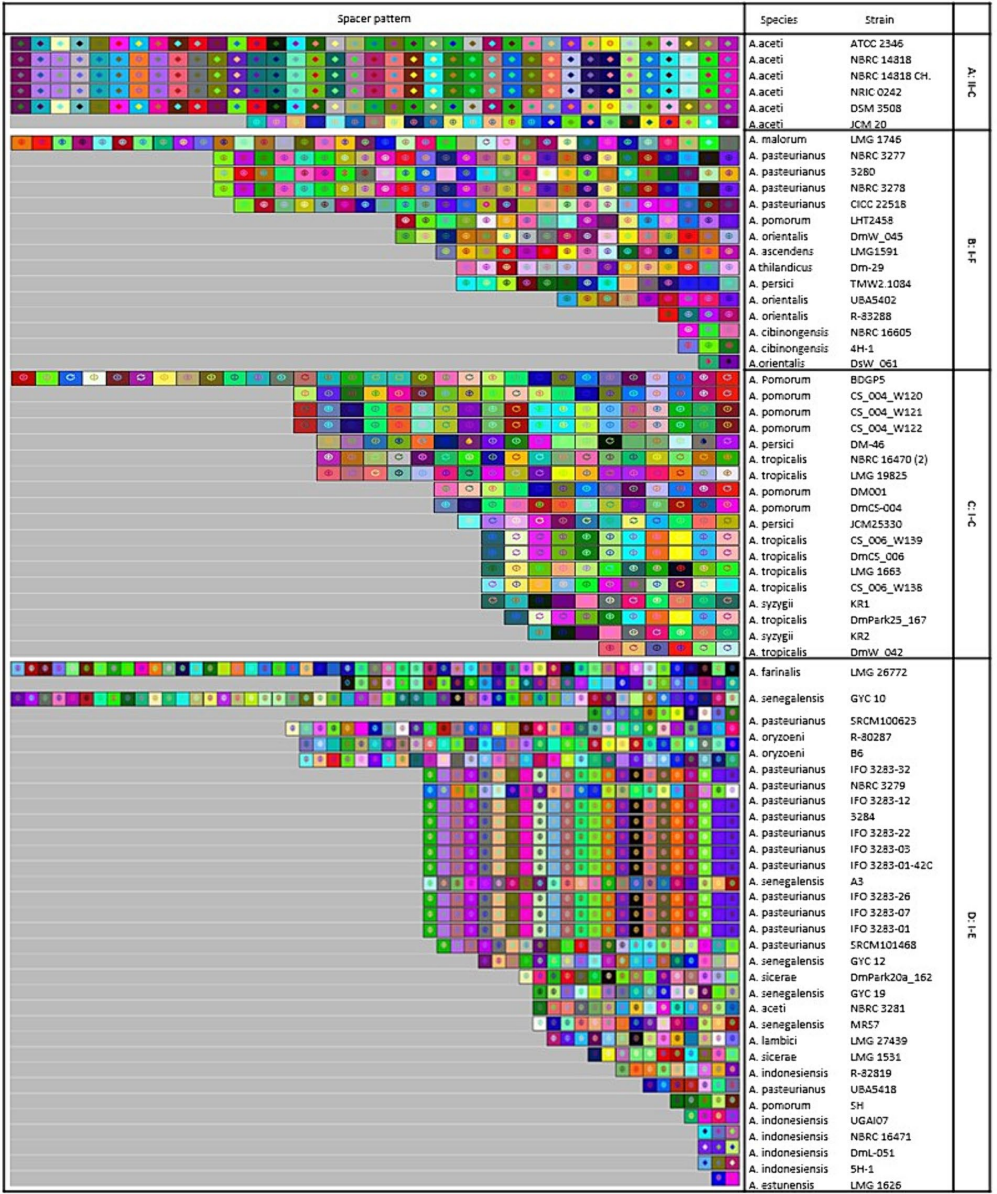
Graphical representation of spacer pattern in subtypes **(A)** II-C; **(B)** I-C; **(C)** I-E; and **(D)** I-F CRISPR-Cas systems. Gray squares containing an “X” represent no spacers. Each color combination represented a unique spacer sequence based on the nucleotide sequences. The newly and earliest acquired spacers are shown on the left and right sides, respectively.

The study focused on examining the evolutionary trajectories of Acetobacter species and its strains when exposed to external genetic material such as plasmids. This was accomplished by analyzing the acquisition and deletion events of spacers. The spacers were arranged in a survey, starting from the ancestral end (right) and ending with the most recently acquired end (left). Each colour combination represented a distinct spacer sequence based on the nucleotide sequences (Figure 6).

In subtype II-C, strains including NBRC 14818, NBRC 14818, and NRIC 0242 shared a completely homogeneous pattern of deletion, acquisition, and composition events of spacers in the evolutionary period (Figure 6A). Similarly, in subtype I-C, *A. pomorum* strains including CS_004_W121 and CS_004_W122, as well as, *A. tropicalis* strains including CS_006_W139 and DmCS_006 shared a completely similar pattern of composition, acquisition, and deletion events of spacers in the evolutionary period (Figure 6B). Several strains from I-E subtype had longer pattern of composition, acquisition, and deletion events compared to other strains. For example, LMG 26772, belonging to *A. farinalis,* had the longest spacer pattern, indicating recent evolutionary events under invasive DNA pressure. In subtype I-E, IFO 3283–32, IFO 3283–12, IFO 3283–22, IFO 3283–03, IFO 3283–01, IFO 3283–07, IFO 3283–26, IFO 3283–01-42C, and 3,284 strains of *A. pasteurianus* had a similar pattern of spacers evolutionary events (Figure 6C). There was a high diversity in the composition and acquisition events of subtype I-F spacers. Only two strains of *A. pasteurianus* including NBRC 3277 and NBRC 3278 showed a completely similar pattern in terms of all indicators, while other strains had unique patterns (Figure 6D).

Strains with a similar pattern of spacers may have originated from a shared ancestor. Therefore, we deduced that strains with similar patterns originated from the same ancestral origin. All strains of subtype I-E showed common lineages originating from different ancestors. It has been demonstrated that strains with a similar pattern of spacers are likely to have been exposed to the same environment and have originated from a shared ancestor (Long et al., 2019). However, in a study conducted by Panahi et al. (2022), examining the sources of isolated strains with different CRISPR-Cas subtypes no significant correlation was found between the subtype of the CRISPR/Cas system and the habitats from which the strains were isolated. Nonetheless, they did find that some strains isolated from similar environments had the same pattern of spacers. These findings suggest that strains with a similar spacer pattern were initially exposed to the same environment, and strains with common lineages diverged after originating from the same ancestral source.

### Spacers homology to plasmid

**Figure 7:**
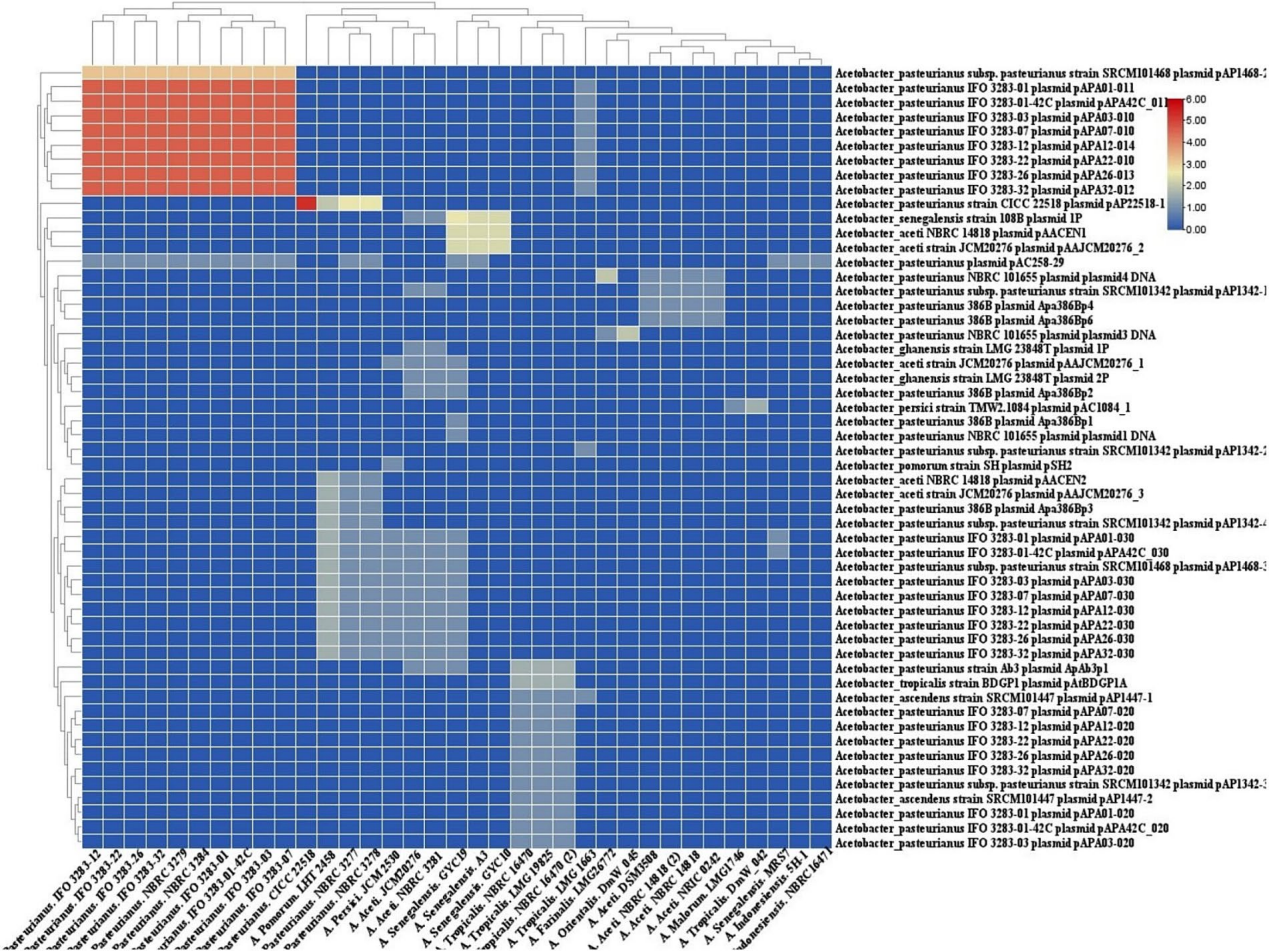
Cluster analysis of homology of spacer in Acetobacter strains targeting with. Acetobacter plasmids. Each box showing the presence of target phages and the color range indicates the frequency of corresponding targets. Blue to red indicates the low number to high number.

The homology between CRISPR spacers and foreign DNA elements such plasmid (Figure 7) sequences can facilitate the transmission of immune information of strains, retrieval of records of encountered threats, and elimination of invasive DNA (Panahi et al., 2023).

In the context of Acetobacter plasmids, the predicted CRISPR-Cas systems in the strains of *A. pasteurianus* target a varying number of Acetobacter plasmids. The subtype I-E targets the most divergent foreign plasmids, while strains with CRISPR-Cas systems I-F and II-C display the lowest diversity of targeting plasmids.

The homology between CRISPR spacers and foreign DNA elements, such as plasmid sequences, plays a crucial role in the immune response of prokaryotes. CRISPR-Cas systems defend against viruses and plasmids by storing short DNA segments of the invader, known as spacers, in the CRISPR array as immunological memories (McGinn and Marraffini, 2016). When a bacterial cell encounters a new foreign DNA threat, new spacer sequences matching the genome of the invading DNA are added to the 5′ end of the CRISPR array (Hille and Charpentier, 2016). This process facilitates the transmission of immune information between strains, retrieval of records of encountered threats, and elimination of invasive DNA. The acquisition of foreign DNA spacers is a vital step in CRISPR-Cas immunity and is achieved through the incorporation of new spacers into the host’s CRISPR array, guided by the host’s endogenous acquisition complex and phage-derived Cas4. The spacers, some of which are homologous to genome segments of viruses and other parasitic genetic elements, are employed as part of guide RNAs to recognize and specifically inactivate the target genomes (Shmakov et al., 2020).

## Conclusion

we analyzed the available genomes of Acetobacter species to assess the occurrence and diversity of CRISPR/Cas systems. The Acetobacter strains were categorized into two groups: those with complete CRISPR/Cas systems, which contained repeat/spacer arrays flanked by Cas genes, and those with orphan CRISPR/Cas systems, which lacked Cas genes. Notably, among the Acetobacter species, *A. pasteurianus* showed the highest occurrence of complete CRISPR/Cas systems, whereas *A. malorum* had the lowest. Overall, the occurrence rate of complete CRISPR/Cas systems in Acetobacter was significantly higher than previously reported rates for other bacteria. The distribution of CRISPR-Cas systems varied significantly among Acetobacter species. *A. pomorum* stood out as the most diverse species, harboring three different subtypes (I-C, I-F, and I-E). The study also highlighted that the presence and prevalence of CRISPR-Cas systems are influenced by both genetic and environmental factors, with strains from high-temperature environments showing higher occurrence rates. Our findings suggest that the structural stability of CRISPR repeats, influenced by factors like stem length and GC pair content, is crucial for the efficiency of CRISPR interference. Subtype II systems, which exhibited the lowest minimum free energy (MFE) and stability, were particularly notable.

Moreover, the analysis revealed distinct patterns of spacer composition, acquisition, and deletion among different CRISPR-Cas subtypes, with some strains showing homogeneous patterns indicative of shared ancestry and environmental exposure. Specifically, subtype II-C strains like NBRC 14818 showed consistent spacer events, while subtype I-E strains exhibited the most diverse and extensive spacer patterns. The study also highlighted the role of spacer homology to plasmid sequences in the immune response, with subtype I-E targeting the most diverse foreign plasmids. These findings suggest that spacer patterns provide insights into the evolutionary history and environmental adaptation of Acetobacter strains, with similar patterns indicating common ancestral origins and environmental pressures.

## Data availability statement

Publicly available datasets were analyzed in this study. This data can be found here: https://www.ncbi.nlm.nih.gov/genome/?term=Acetobacter.

## Author contributions

SG: Writing – original draft, Validation, Resources, Investigation, Formal analysis, Data curation, Conceptualization. BP: Writing – review & editing, Visualization, Validation, Supervision, Project administration, Methodology.

## Footnotes

^1^ https://www.ncbi.nlm.nih.gov/

^2^ https://omics.informatics.indiana.edu/CRISPRone/denovo.php

^3^ https://weblogo.berkeley.edu/logo.cgi
